# Geographic Variation in the Structure of Kentucky’s Population Health Systems: An Urban, Rural, and Appalachian Comparison

**DOI:** 10.13023/jah.0203.04

**Published:** 2020-07-19

**Authors:** Rachel Hogg-Graham, Angela Carman, Glen P. Mays, Pierre Martin Dominique Zephyr

**Affiliations:** University of Kentucky, rachel.hogg@uky.edu; University of Kentucky, angela.carman@uky.edu; Colorado School of Public Health, University of Colorado, glen.mays@cuanschutz.edu; University of Kentucky, dominique.zephyr@uky.edu

**Keywords:** Appalachia, public health, organization of care, rural health, population health, delivery of care, geographic variation

## Abstract

**Introduction:**

Research examining geographic variation in the structure of population health systems is continuing to emerge, and most of the evidence that currently exists divides systems by urban and rural designation. Very little is understood about how being rural and Appalachian impacts population health system structure and strength.

**Purpose:**

This study examines geographic differences in key characteristics of population health systems in urban, rural non-Appalachian, and rural Appalachian regions of Kentucky.

**Methods:**

Data from a 2018 statewide survey of community networks was used to examine population health system characteristics. Descriptive statistics were generated to examine variation across geographic regions in the availability of 20 population health activities, the range of organizations that contribute to those activities, and system strength. Data were collected in 2018 and analyzed in 2020.

**Results:**

Variation in the provision of population health protections and the structure of public health systems across KY exists. Urban communities are more likely than rural to have a comprehensive set of population health protections delivered in collaboration with a diverse set of multisector partners. Rural Appalachian communities face additional limited capacity in the delivery of population health activities, compared to other rural communities in the state.

**Implications:**

Understanding the delivery of population health provides further insight into additional system-level factors that may drive persistent health inequities in rural and Appalachian communities. The capacity to improve health happens beyond the clinic, and the strengthening of population health systems will be a critical step in efforts to improve population health.

## INTRODUCTION

Residents of rural communities typically have poorer health outcomes and complex needs such as geographic isolation, lower socioeconomic status, and lower education attainment, making efforts that seek to address medical needs alone largely insufficient.^1^ Strengthening the delivery of public health and social services as a mechanism to address population health may be one strategy to improve health outcomes across the U.S. and reduce geographic inequities.^2^,^3^

Population health activities typically include community-level protections and can range from the monitoring of adverse health events and outbreaks to community health needs assessments and implementation plans, health inspections, and health education and policy development to promote behavior change and prevention (e.g., smoking cessation and or tobacco taxation).^4^ Key in many population health activities is the development of multisector relationships that reduce fragmentation and increase efficient delivery of services.^5^,^6^ The development of multisector systems of care and delivery of public health services may be more difficult in rural communities. To provide insight into this topic, this study examines geographic differences in key characteristics of population health systems, including the number of population health activities implemented in the community and the range of multisector contributions to population health. This study focuses on geographic variation among urban, rural non-Appalachian, and rural Appalachian communities in the state of Kentucky (KY), with the hypothesis that rural Appalachian communities will be the least-well served of these areas.

Recent research by Harris et al.^7^ highlighted the “double disparity” faced by many rural communities resulting from consistently poor health behaviors and chronic underfunding of public health. Public health agencies in rural areas are less likely to provide the same number of population health services as their urban counterparts, have fewer employees, and are often working with a limited set of community partners. These geographic differences in population health capacity can lead to inadequate population health protections in rural communities, potentially exacerbating adverse health outcomes in areas that are already medically underserved.

Research examining geographic variation in the structure of population health systems is continuing to emerge, and most evidence that currently exists divides systems by urban and rural designation. However, rural is not the same from region to region and can vary within states. For example, Appalachia is one geographic area that crosses state lines and has been identified as a region with a long history of poverty and poor health outcomes.^8^,^9^ Appalachian counties typically have higher mortality and morbidity rates and face significant medical and social resource shortages compared to non-Appalachian counties, making it possible they also have limited population health capacity. Very little is understood about how being rural and Appalachian affects the strength and structure of the population health system. Examining geographic variation in population health systems beyond urban and rural designation may provide insight into additional resource and system constraints that affect underserved areas and lead to poor health outcomes.

## METHODS

A cross-section of data from 2018 on Kentucky’s communities from the National Longitudinal Survey of Public Health Systems (NALSYS) was used to examine health and social services network characteristics and how those differ across geographic regions. NALSYS surveys a stratified random sample of the nation’s 3000 local public health officials. Respondents report whether or not a set of core population health activities is provided in the community and then select the organizations that participate in each activity (Table 1 has a full list of activities). The number of activities selected and the reported organization contributions are used to generate proportion measures that identify the percent of activities provided in a community and the percent of those activities that each organizational sector engages in delivering. The unit of analysis is the local public health jurisdiction. All KY jurisdictions (n=61) were included in the 2018 NALSYS sample, providing a unique opportunity to examine variation in the provision of population health protections and the structure of population health systems across the state. The University of Kentucky IRB determined this study exempt.

To examine geographic variation, communities were coded as urban or rural using Rural–Urban Continuum (RUCA) Codes. RUCA codes geographically define communities using a numbered classification system that measures urbanization, population density, and commuting.^10^ Although there are a number of ways to define rurality, RUCA codes were selected in accordance with the definition used by the Federal Office of Rural Health Policy. Rural jurisdictions were further stratified by identifying communities in the Appalachian region to create three geographic comparison groups: urban (n=20), rural non-Appalachia (n=15), and rural Appalachian (n=26).

Kentucky has a mix of single and multi-county public health jurisdictions. Of the 14 multi-county jurisdictions, three were composed of all Appalachian counties, seven of all non-Appalachian, and four had a mix. For urban and rural designation, six were all rural, two were all urban, and six were mix. Jurisdictions with counties in multiple categories were coded based on where the majority of the population resides. Three counties in Kentucky’s Appalachian region are urban. For the purpose of this paper, these counties were coded urban to keep the rural Appalachian category distinct.

Descriptive and bivariate analyses were used to examine variation across geographic regions in the availability of 20 population health activities, the range of organizations that contribute to those activities, and population health system capital. A t-test was used to examine differences in activities and organizations and a chi-squared for population health capital. Population health system capital measures the strength of the system using a three-group classification. Communities were classified as having comprehensive, conventional, or limited levels of system capital using the results of a cluster analysis performed with measures of (1) the proportion of 20 recommended public health activities implemented in the community and (2) the array of organizations contributing to each activity in the community. Numeric thresholds for distinguishing each class were identified using latent class analysis.^11^ Comprehensive communities are those with the highest range of activities provided with the broadest network of multisector organizations contributing to those activities. Limited reflects less multisector engagement and a smaller scope of activities, with conventional falling in the middle. Data were collected in 2018 and analyzed in 2020. Analyses were conducted using Stata version 16 (College Station TX).

## RESULTS

Substantial variation in the delivery of population health activities across KY exists (Table 1). On average, systems in urban areas provide a higher number of population health activities than their rural non-Appalachian and rural Appalachian counterparts. Variation in the magnitude of differences in the availability of activities across geographic regions also exists. For example, no significant difference in the level at which regions are investigating adverse health events exists. At the same time, 100% of urban communities reported conducting a community health needs assessment while only 64.7% of rural non-Appalachian and 61.5% of rural Appalachian communities are implementing this activity.

**Figure 1:**
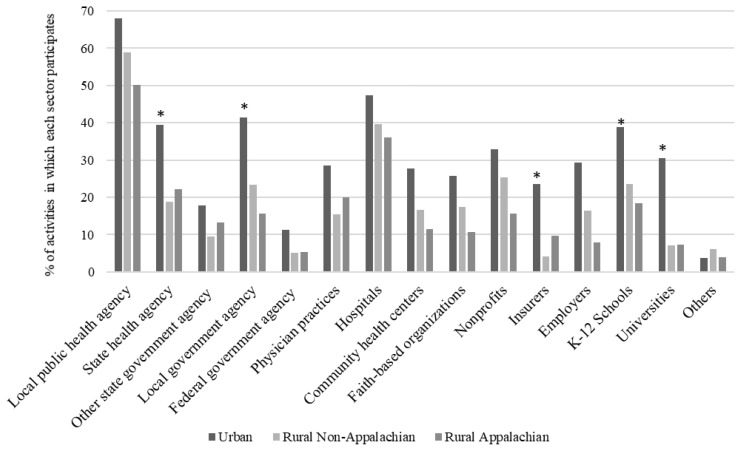
Organization contributions to population health across urban, rural non-Appalachian, and rural Appalachian public health jurisdictions * Statistically different from rural non-Appalachian communities, t-test p<0.05, Bonferroni adjustment for multiple comparisons. Notes. Values represent the portion of communities that responded “yes” that organization contributes to the activity (dichotomous yes/no question), stratified by geographic region.

On average, multisector contributions to population health are higher in urban communities, with local public health agencies, hospitals, local government agencies, state health agencies, K–12 schools, and nonprofits participating in the most activities (Figure 1). A similar pattern exists in rural non-Appalachian and Appalachian communities, although overall contributions are at a lower level than their urban counterparts. Similar to the implementation of population health activities across geographic regions, variation in the magnitude of organization total contribution exists. Universities and insurers participate in over 20% of activities in urban communities, compared to less than 10% in rural (p<0.05).

These findings also indicate variation in the implementation of activities between rural regions. On average, rural non-Appalachian communities are providing activities at a higher rate than rural Appalachian. Rural Appalachian communities are less likely to develop policies that address priorities in community health plans, monitor and improve the implementation of health programs and policies, and provide community health information to the media (p<0.05). However, it is worth highlighting a small set of activities, including the analysis of data and preventative services use, providing community health information to elected officials, and the linking of individuals to needed health and social services, that Appalachian communities are implementing at a slightly higher rate, although not statistically significant. Similarly, physician organizations, state health agencies, insurers, and other state agencies participate in more activities in rural Appalachian communities than rural non-Appalachian.

**Figure 2:**
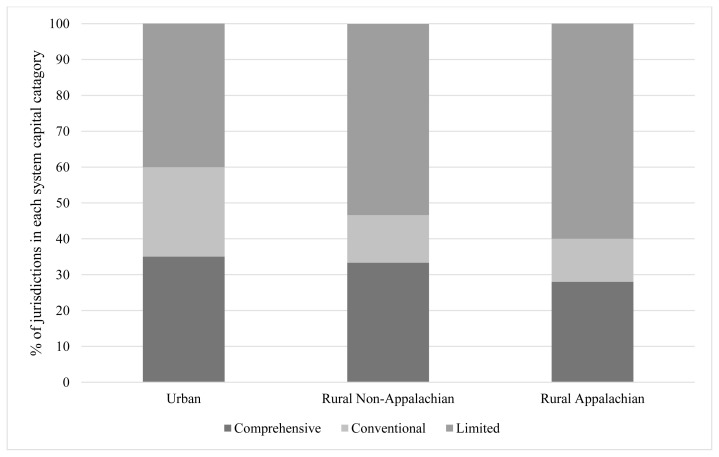
Population health system capital across urban, rural non-Appalachian, and rural Appalachian public health jurisdictions Note: Chi-squared test did not return significant results.

The majority of systems in KY have limited system capital (Figure 2). This pattern was consistent across regions, with 60% of rural Appalachian communities ranking as limited, 40% of urban, and 53% of rural non-Appalachian. Comprehensive is the second most prevalent type of system capital in the state, with 35% of urban communities, 33% of rural non-Appalachian, and 28% of rural Appalachian falling into this category.

## IMPLICATIONS

This study suggests that substantial geographic variation in the provision of population health protections and the structure of population health systems across the state of KY exists. Urban communities are more likely than rural to have a comprehensive set of population health protections delivered in collaboration with a diverse set of multisector partners. Rural Appalachian communities face additional limited capacity in the delivery of population health activities, compared to other rural communities in the state.

Hospitals and local public health agencies play the most prominent role throughout systems in KY, suggesting they may be central players in strengthening capacity, particularly in rural and Appalachian systems that have limited engagement from other sectors. Low rates of assurance and evaluation activities across all geographic regions points to important gaps in activities critical to sustained population health improvement. Similarly, the majority of population health systems across the state have limited system capital, meaning they provide a smaller scope of population health activities with less breadth in the range of multisector organizations that contribute to implementation. Recent research has shown that, as system capital increases, preventable mortality decreases, suggesting that opportunity for health outcome improvement exists by strengthening capacity in limited and conventional systems.^6^

Although not statistically significant, it is important to note that rural Appalachian communities did report slightly higher rates of a select number of activities compared to non-Appalachian rural communities, specifically in assurance and evaluation activities. Additionally, they reported greater engagement of physicians, insurers, and state agencies. These findings may reflect greater health and social needs on the part of rural Appalachian residents and concerted efforts to address needs through multisector engagement and a commitment to public health improvement.

Variation in population health system structure is likely driven by greater health needs, limited availability of community partners, and resource constraints. Rural communities are smaller and often have fewer available organizations to draw on in creating multisector networks. Rural communities are also commonly designated as Health Provider Shortage Areas (HPSAs), meaning they lack an adequate number of clinical providers to meet population need in primary care, dental, and mental health. Public health agencies often help fill those gaps in care by providing basic clinical services to the populations they serve. Current reimbursement structures also favor clinical services, where billing mechanisms like Medicaid and Medicare reimburse for direct services compared to population health funding that is typically grant or local tax dollar based. Lower population density and a focus on agriculture businesses may reduce the availability of local tax dollars in rural areas, with these funds being further limited in Appalachian communities due to the reduction of businesses and population migration following the closing of coalmines. These factors likely contribute to the gaps in population health activities identified in this study and thus the impact of those gaps in areas of greater health needs.

While significant variation in the delivery of population health activities and multisector contributions to these activities exists across KY, understanding variation provides critical information that can aide in building capacity. Using the information on where gaps exist, local public health agencies could start by doing an environmental scan to understand community needs and where priority areas for action exist. Engaging other community organizations is also a crucial step in the process to ensure coordination of efforts across diverse sectors. Models like the Mobilizing for Action through Planning and Partnerships (MAPP) and the Process Framework for Public Health Collaboratives are useful tools to help ensure community assessment is done “with” the community and not just “about.”^4^,^12^ Working to fill the gaps with community partners will naturally increase the number of activities being implemented in a multisector framework and help move the system toward comprehensive capital.

As conversations around the U.S. continue to discuss rural and Appalachian communities as being underserved, these findings highlight an additional area for discussion: Are these communities also population-health and social-service underserved? Although a number of resource and policy initiatives have targeted increasing medical providers and services in rural Appalachian and non-Appalachian communities, health inequities continue to persist. A growing body of research suggests that the delivery of population health through multisector partnerships is a key element in improving health outcomes, and these findings suggests that gaps in these services exist across KY.^6^ Understanding the social factors and systemic issues that influence health and wellbeing are critical factors that need to be addressed by communities. Population health organizations, such as local public health agencies, can act as conveners of the diverse set of organizations needed to address health and social issues. However, it is important to acknowledge that building the capacity to create strong and sustainable multisector population health systems is not easy. Conveners need the time and leadership skills to implement strategies that engage multisector organizations in population health activities. Public health organizations are often asked to engage in, and frequently lead, systems work with little information on how to best carryout that process. Considering how building these systems across diverse geographic regions and in rural and Appalachian areas that are chronically underserved adds additional complexities as communities consider ways to increase population health system capital. Expanding and rethinking programs that target increasing capacity and access to medical providers and services in rural non-Appalachian and Appalachian communities in KY to also include mechanisms that strengthen the population health system will be critical in advancing population health goals.

To the best of our knowledge, this is the first study to examine geographic variation in the provision of population health activities and structure of systems in KY, but this study is not without limitations. This analysis is descriptive and exploratory and should be used to generate ideas for further study. Understanding the causes and consequences of variation in multisector delivery of population health activities would be an important next step in building the evidence base that examines the complex relationship between multisector population health initiatives and health outcomes. Expanding the scope beyond KY to examine differences between other urban, rural non-Appalachian, and Appalachian population health systems would be another important avenue to better understand inequities in population health protections. Understanding the delivery of population health and structure of local public health systems in the state provides further insight into additional system-level factors that may drive persistent health inequities in rural and Appalachian communities. The capacity to improve health happens beyond the clinic, and the strengthening of population health and social service systems of care will need to happen before significant gains in health improvement will be achieved.

**SUMMARY BOX**

**What is already known about the subject?** Research examining geographic variation in the structure of population health systems suggests that differences in capacity exist between rural and urban communities.

**What is added by this report?** This study confirms previous findings and expands the evidence base by identifying significant variation between not only urban and rural communities, but also Appalachian.

**What are the implications?** Insight into additional system-level factors that extend beyond medical capacity provides a starting point for future studies that may wish to examine how limited population health and social service capacity impacts persistent health inequities in rural and Appalachian communities.

## Figures and Tables

**Table 1:** Implementation of population health activities across urban, rural non-Appalachian, and rural Appalachian public health jurisdictions

	Urban	Rural Non-Appalachian	Rural Appalachian
**Number of districts in region (% of districts in KY)**	**20 (33)**	**15 (24)**	**26 (43)**
**Assessment**			
Conduct periodic assessment of community health status and needs	95.0*	66.7	61.5
Survey community for behavioral risk factors	60.0	53.3	40.0
Investigate adverse health events, outbreaks, and hazards	95.0	100.0	96.0
Conduct laboratory testing to identify health hazards and risks	90.0	85.7	84.0
Analyze data on community health status and health determinants	90.0*	57.1	37.5
Analyze data on preventative services use	35.0	13.3	19.2
**Policy and Planning**			
Routinely provide community health information to elected officials	80.0	73.3	88.0
Routinely provide community health information to the public	90.0	73.3	60.0
Routinely provide community health information to the media	95.0	100.0	75.0*
Prioritize community health needs	100.0*	73.3	60.0
Engage community stakeholders in health improvement planning	90.0*	40.0	29.2
Develop a community-wide health improvement plan	90.0	73.3	61.5
Allocate resources based on community health plan	70.0	46.7	24.0
Develop policies to address priorities in community health plan	50.0	73.3	36.0*
Maintain a communication network among health-related organizations	95.0	93.3	80.0
**Assurance and Evaluation**			
Link people to needed health and social services	45.0	35.7	48.0
Implement legally mandated public health activities	100.0	86.7	100.0
Evaluate health programs and services in the community	45.0	33.3	16.0
Evaluate public health agency capacity and performance	26.3	40.0	44.0
Monitor and improve implementation of health programs and policies	47.4	33.3	8.0*

* Statistically different from rural non-Appalachian communities, t-test p<0.05, Bonferroni adjustment for multiple comparisons.

Notes. Values represent the portion of communities that responded “yes” to providing the activity (dichotomous yes/no question), stratified by geographic region.
